# A compound heterozygous case of leukocyte adhesion deficiency type-1 with moderate CD18 expression and severe disease

**DOI:** 10.70962/jhi.20250124

**Published:** 2025-08-22

**Authors:** Jeremy Richard Brozyna, Rosemary Moak, Loretta Modica Parker, Thomas Prescott Atkinson

**Affiliations:** 1 Division of Allergy and Immunology, Department of Pediatrics, University of Alabama at Birmingham, Birmingham, AL, USA; 2 Division of Pediatric Hematology/Oncology, Department of Pediatrics, University of Alabama at Birmingham, Birmingham, AL, USA

## Abstract

A child with severe leukocyte adhesion deficiency type-1 (LAD-1) was found to have compound heterozygous ITGB2 mutations. Despite moderate CD18 expression, the patient experienced life-threatening infections, demonstrating that protein levels do not always correlate with clinical severity.

## Teaser

Recurrent pneumonias and skin infections with persistent leukocytosis are concerning for leukocyte adhesion deficiency type-1. Intermittently normal neutrophil counts can be seen with partial deficiency. Compound heterozygotes, such as the patient described, may be predisposed for severe disease despite exhibiting moderate integrin expression profiles.

Leukocyte adhesion deficiencies are an autosomal recessive group of immunodeficiency disorders in which leukocytes and particularly neutrophils are impaired in their ability to adhere to the endothelium and migrate to sites of infection. Leukocyte adhesion deficiency type-1 (LAD-1) is characterized by loss-of-function variants in *ITGB2*, the gene coding for CD18—the heavy chain in the β2 integrin heterodimer. Defects in this gene can lead to patients suffering from recurrent and severe bacterial and fungal infections, delayed umbilical cord separation, and omphalitis (1, 2). Additional findings include the inability to form pus, poor wound healing, and persistent neutrophilia (3). Mortality rate is high and correlates with the degree of decreased CD18 expression. Hematopoietic stem cell transplantation is curative.

A 4-mo, 19-day-old male with history of full-term delivery, eczema, seborrheic dermatitis, two prior hospitalizations for pneumonias (one requiring intubation), and bilateral otitis media with left tympanic membrane rupture presented to the emergency department with fever (38.8°C). There was no family history of immunodeficiency or recurrent infections, and no suspected consanguinity. Physical examination revealed balanitis and scrotal cellulitis without erythema. He was found to have leukocytosis of 37,140/mm^3^ (reference range: 6,900–15,700/mm^3^) with neutrophilia of 17,780/mm^3^ (1,400–6,400/mm^3^) and lymphocytosis of 14,990/mm^3^ (2,800–8,300/mm^3^). At 3 mo, 28 days old (about 3 wk prior), his immunoglobulins had included elevated IgG 843 mg/dl (196–558 mg/dl) and IgM 161 mg/dl (27–101 mg/dl), as well as an IgA of 42 mg/dl (4.4–73 mg/dl) and IgE of 32.6 IU/ml (0.18–3.76 IU/ml). Titers to diphtheria and tetanus after routine scheduled 2-mo vaccinations and prior to 4-mo vaccinations were protective at 0.37 IU/ml (≥0.10 IU/ml) and 0.60 IU/ml (≥0.10 IU/ml), respectively. Pneumococcal titers following one pneumococcal conjugate vaccine-20 (PCV20) revealed seven serotype-specific titers in the protective range at ≥1.3 μg/ml (see Table 1). For his balanitis and scrotal cellulitis, he was treated with intravenous clindamycin that was transitioned to oral therapy.

**Table 1.** Timeline of clinical events for an infant with *ITGB2* c.850G>A (p.Gly284Ser) and *ITGB2* c.314T>C (p.Leu105Pro) variants concerning for moderate LAD-1 (by integrin expression profile)

Age	Clinical presentation	Laboratory data^a^
1 mo and 23 days old	Bilateral pneumonia requiring intubation (as well as re-intubation)	WBC 30,080/mm^3^ (6,900–15,700/mm^3^) ANC 20,980/mm^3^ (1,400–6,400/mm^3^) ALC 6,340/mm^3^ (2,800–8,300/mm^3^) Positive PCR for mycoplasma, RSV, rhinovirus/enterovirus and coronavirus.
2 mo and 11 days old	Peak leukocytosis and neutrophilia on same day as re-intubation for respiratory failure due to pneumonia (same hospitalization for aforementioned pneumonia/intubation above)	WBC 69,250/mm^3^ ANC 63,710/mm^3^ Pseudomonas identified on sputum culture during re-intubation
3 mo and 28 days old	Left lower lobe pneumonia, bilateral otitis media including left tympanic membrane rupture, tinea capitis. Rehospitalization compared to above	WBC 51,710/mm^3^ (6,900–15,700/mm^3^) ANC 33,460/mm^3^ (1,400–6,400/mm^3^) ALC 11,550/mm^3^ (2,800–8,300/mm^3^) IgG 843 mg/dl (196–558 mg/dl) IgM 161 mg/dl (27–101 mg/dl) IgA 42 mg/dl (4.4–73 mg/dl) IgE 32.6 IU/ml (0.18–3.76 IU/ml) Diphtheria IgG 0.37 IU/ml (≥0.10 IU/ml) Tetanus IgG 0.60 IU/ml (≥0.10 IU/ml) Pneumococcal IgG titers following one PCV20 revealed seven serotype-specific titers in the protective range at ≥1.3 μg/ml
4 mo and 19 days old	Balanitis and scrotal cellulitis without erythema	WBC 37,140/mm^3^ (6,900–15,700/mm^3^) ANC 17,780/mm^3^ (1,400–6,400/mm^3^) ALC 14,990/mm^3^ (2,800–8,300/mm^3^) Flow cytometry: CD11b expression of 16% and CD18 expression of 6% compared with healthy control subjects
4 mo and 26 days old	(At baseline health status)	WBC 26,430/mm^3^ (6,900–15,700/mm^3^) ANC 5,860/mm^3^ (1,400–6,400/mm^3^) Immature granulocytes 90/mm^3^; (0–50/mm^3^) ALC 17,920/mm^3^ (2,800–8,300/mm^3^)
6 mo and 6 days old	(At baseline health status)	WBC 22,130/mm^3^ (5,980–13,510/mm^3^) ANC 3,620/mm^3^ (1,190–7,210/mm^3^) ALC 16,600/mm^3^ (1,560–7,830/mm^3^) AEC 620/mm^3^ (30–350/mm^3^).
6 mo and 27 days old	(At baseline health status)	WBC 27,850/mm^3^ (5,980–13,510/mm^3^). ANC 4,310/mm^3^ (1,190–7,210/mm^3^). ALC 21,530/mm^3^ (1,560–7,830/mm^3^). AEC 290/mm^3^ (30–350/mm^3^) CD3^+^ cells 13,182/mm^3^ (2,500–5600/mm^3^); 61% of lymphocytes (49–76%) CD4^+^ cells 9,221/mm^3^ (1,800–4,000/mm^3^); 43% of lymphocytes (31–56%) CD8^+^ cells 2,577/mm^3^ (590–1,600/mm^3^); 12% of lymphocytes (12–24%) CD19^+^ cells 6,116/mm^3^ (430–3,000/mm^3^); 28% of lymphocytes (14–37%) CD16^+^ cells 2,452/mm^3^ (170–830/mm^3^); 11% of lymphocytes (3–15%)

AEC, absolute eosinophil count; ALC, absolute lymphocyte count; ANC, absolute neutrophil count; WBC, white blood cell count.

a Reference ranges are shown in parentheses.

He had recurrent anemia believed to be secondary to other disease processes and was never thrombocytopenic. During admission for his initial pneumonia at 1 mo and 23 days of age, microbiological analysis was positive for *Pseudomonas aeruginosa* on sputum culture as well as *Mycoplasma pneumoniae*, respiratory syncytial virus (RSV), rhinovirus/enterovirus and coronavirus by PCR, and the patient required intubation (as well as re-intubation) due to respiratory failure. The patient had a peak leukocytosis of 69,250/mm^3^ with neutrophilia of 63,710/mm^3^ at 72 days of age, the same day as he underwent re-intubation. The second admission for pneumonia at 3 mo and 28 days of age was associated with a positive rhinovirus/enterovirus PCR test, though the patient did not require intubation during this illness.

Flow cytometry analysis at 4 mo and 19 days of age demonstrated CD11b expression of 16% and CD18 expression of 6% compared with healthy control subjects, consistent with LAD-1 with partial expression of leukocyte β2 integrins. The patient was started on oral prophylactic trimethoprim-sulfamethoxazole 4 mg/kg by trimethoprim component, itraconazole 3.7 mg/kg, and amoxicillin 40 mg/kg daily. Follow-up complete blood count at 4 mo, 26 days of age continued to exhibit leukocytosis of 26,430/mm^3^ (reference range: 6,900–15,700/mm^3^), although with neutrophil counts within normal limits (5,860/mm^3^; 1,400–6,400/mm^3^), left shift (immature granulocytes 90/mm^3^; 0–50/mm^3^), and lymphocytosis of 17,920/mm^3^ (2,800–8,300/mm^3^). Leukocytosis with lymphocytosis and neutrophils within normal limits were persistent at 6 mo, 6 days old, as well as 6 mo, 27 days old, while the patient was in his baseline normal state of health (Table 1). Pathology peripheral smear review was consistent with leukocytosis with reactive lymphocytes.

Flow cytometry in baseline health status at 6 mo and 27 days old demonstrated 61% (of lymphocytes) CD3^+^ cells (reference range: 49–76%), 43% CD4^+^ cells (31–56%), 12% CD8^+^ cells (12–24%), 28% CD19^+^ cells (14–37%), and 11% CD16^+^ cells (3–15%). There was normal immunophenotyping without blasts, monotypic B cell population, or a phenotypically aberrant T cell population.

Genetic testing revealed *ITGB2* c.850G>A (p.Gly284Ser) pathogenic variant and *ITGB2* c.314T>C (p.Leu105Pro) likely pathogenic variant. The *ITGB2* c.850G>A (p.Gly284Ser) pathogenic variant in exon 7 has been described in homozygous and heterozygous genetic variants, including on the opposite chromosome (trans-configuration) to a known different pathogenic variant, as well as in segregation with affected family members to cause LAD-1 in patients (2, 3). It substitutes a neutral polar amino acid for a neutral nonpolar amino acid within the N-terminal extracellular β integrin domain, which is critical for ligand binding. This substitution does not support CD11/CD18 cell surface expression in *in vitro* transfection studies (4). It is rare in the general population with a gnomAD allele frequency of 1.14 × 10^−4^ (rs137852616). The *ITGB2* c.314T>C (p.Leu105Pro) variant is present in the gnomAD population database with a frequency of 4.52 × 10^−5^ (rs145851783). A homozygous c.314T>C patient has been reported, as well as a likely heterozygous patient also with partial deletion of exon 7 (c.742-163_768del), both with absent expression of CD11/CD18 (<1%) (5). Both of these reported patients, 2- and 11-year-old African American boys, presented with recurrent and severe pyoderma gangrenosum.

The patient presented herein is also African American, however he did not have pyoderma gangrenosum but presented with recurrent pneumonia and scrotal cellulitis and at a younger age than the aforementioned patients with the *ITGB2* c.314T>C (p.Leu105Pro) variant. This report adds additional information to the pathogenicity of the p.Leu105Pro ITGB2 variant, including a different clinical presentation more aligned to a phenotype including invasive bacterial infections. Moreover, mortality of severe LAD-1 (<2% of CD18 expression by neutrophils) has been reported to be 75% by 2 years of age, whereas patients with moderate LAD-1 (2–30% CD18 expression) typically survive childhood (2). Despite being classified as moderate LAD-1 given 16% CD11b and 6% CD18 expression, the patient reported has had multiple pneumonias including requiring intubation, as well as balanitis and scrotal cellulitis within the first year of life. Thus, his infectious history is more concerning than what would be expected from previous reports given his genetic predisposition and integrin expression profile.

Several reports have detailed a severe clinical phenotype despite either severe or moderate CD18 expression profiles of LAD-1 patients. These patients were noted to be compound heterozygotes, which may be associated with a lack of consanguinity compared with homozygotes (1, 2, 3). It has been purported that compound heterozygotes may have a severe clinical phenotype despite their level of CD18 expression, given they harbor two different defective alleles, which may be responsible for distinct molecular interactions or function (1). Sun et al. detailed six compound heterozygotes, four of whom had moderate CD18 expression (2.5–6.5%), although each suffered from severe infections early in life (≤15 mo of age). Similarly, the patient described herein has 6% CD18 expression, is a compound heterozygote, and has suffered from severe infections early in life. It may be that compound heterozygotes with either moderate or severe CD18 expression profiles have propensity for a more severe clinical phenotype.

The patient presented here had developed a leukemoid reaction during periods of illness with marked neutrophilia. However, on the most recent complete blood counts while he was at baseline, he had lymphocytosis with neutrophils in the normal range. Flow cytometry analysis of his lymphocytes revealed a normal distribution of subsets. Nevertheless, his severe infections and extreme neutrophilia suggested a diagnosis of leukocyte adhesion deficiency, which was confirmed with flow cytometry and genetic testing.

Diagnoses comprising cellulitis, skin ulcerations, omphalitis, and pneumonia have been reported to be in similar frequencies between moderate and severe LAD-1, however moderate LAD-1 is associated with twice the frequency of pyoderma gangrenosum compared with severe LAD-1 (5). The reason for this association is unclear. While the patient discussed had a history of *P. aeruginosa* identified on sputum culture as a cause of pneumonia, he did not have delayed umbilical cord separation, gastrointestinal disease, or ulcerative skin disease including pyoderma or ecthyma gangrenosum. Because of the severity of his infections and the availability of an HLA-identical sibling (12/12-matched sister with normal CD11b/CD18 expression), the decision was made to proceed with hematopoietic stem cell transplant.

In summary, clinical history of recurrent pneumonias and skin infections with persistent leukocytosis are concerning for LAD-1. Intermittently normal absolute neutrophil counts can be seen with partial deficiency. The *ITGB2* c.850G>A (p.Gly284Ser) pathogenic variant is rare in the population and well described in literature for causing severe LAD-1. The *ITGB2* c.314T>C (p.Leu105Pro) variant is also rare and has an undefined pathogenicity that may have a predisposition for cutaneous disease. These combined mutations should be evaluated for possible LAD-1, especially with a significant infectious history. Compound heterozygotes, such as the patient described herein, may be predisposed for severe disease despite exhibiting moderate integrin expression profiles.

## Key message

Leukocyte adhesion deficiency may present with variable severity, including severe clinical outcomes, despite average integrin expression profiles.

## Informed consent

Local ethics board approval was not required; the patient’s family consented and signed a medical care release for publication. The authors would like to thank the patient and their family for their contribution.

## Data Availability

The data are available in the article and from the corresponding author upon reasonable request.
